# The Immunologic Role of IL-17 in Psoriasis and Psoriatic Arthritis Pathogenesis

**DOI:** 10.1007/s12016-018-8702-3

**Published:** 2018-08-14

**Authors:** Andrew Blauvelt, Andrea Chiricozzi

**Affiliations:** 1 Oregon Medical Research Center, 9495 SW Locust St, Suite G, Portland, OR, 97223 USA; 2 Dermatology Unit, Department of Clinical and Translational Medicine, University of Pisa, Pisa, Italy

**Keywords:** Psoriasis, IL-17A, IL-17F, IL-17 receptor A, Innate immunity, Adaptive immunity

## Abstract

Psoriasis is a chronic, immune-mediated, inflammatory disease that is pathogenically driven by proinflammatory cytokines. This article reviews the immunologic role of interleukin (IL)-17, the major effector cytokine in the pathogenesis of psoriatic disease, along with the rationale for targeting the IL-17 cytokine family (IL-17A, IL-17F, and IL-17 receptor A) in the treatment of psoriasis and psoriatic arthritis. Emerging evidence indicates that major sources of IL-17A in patients with psoriatic disease are mast cells, γδ T cells, αβ T cells, and innate lymphoid cells in lesional skin and synovial fluid. Within the skin and joints, IL-17A acts on cellular targets, including keratinocytes, neutrophils, endothelial cells, fibroblasts, osteoclasts, chondrocytes, and osteoblasts, to stimulate production of various antimicrobial peptides, chemokines, and proinflammatory and proliferative cytokines, which, in turn, promote tissue inflammation and bone remodeling. The critical importance of the IL-23/IL-17A axis to the pathogenesis of psoriatic disease has resulted in many new biologic treatments targeting these cytokines. These biologics dramatically improve skin and joint symptoms in patients with moderate-to-severe psoriasis and psoriatic arthritis.

## Introduction

Psoriasis is a chronic, immune-mediated, inflammatory disease in which genetic and epigenetic changes result in a disease phenotype characterized by altered immune function, keratinocyte activation and hyperproliferation, and the development of erythematous, indurated, scaly plaques [1–4]. Psoriasis is driven by T cell activation associated with the secretion of proinflammatory cytokines, including tumor necrosis factor-α (TNF-α), interleukin (IL)-17A, IL-22, and interferon IFN-γ [1, 5]. The IL-23/IL-17 immunologic pathway plays an especially important role in promoting disease onset and perpetuation. Data from in vitro and clinical studies indicate that IL-17A, a critical effector cytokine in this pathway, principally drives changes within affected tissues [6–11].

Direct evidence supporting the central role of IL-17A in psoriasis includes upregulation of *IL-17A* and related genes in lesional and non-lesional skin of patients with psoriasis and production of IL-17A by cells associated with psoriasis [6, 8, 12]. In an in vitro study using reconstituted human epidermal sheets, IL-17A stimulated greater transcriptional activation than IL-22 or IFN-γ, correlating with the psoriasis transcriptome [13]. IL-17 also increases expression of the antimicrobial peptide LL37, a psoriasis autoantigen that promotes production of proinflammatory cytokines, and C-X-C motif chemokine ligand 1 (CXCL1) [14, 15]. This, in turn, drives expansion of ADAMTS-like protein 5 (ADAMTSLP5), another psoriasis autoantigen, causing additional expression of IL-17A and IFN-γ [15, 16].

In addition to IL-17A, the IL-17 family consists of five other members (IL-17B-F) [17–23]. Within this family, IL-17A, IL-17C, and IL-17F are implicated in psoriasis pathogenesis as their expression is increased up to eightfold in psoriatic lesions [6, 24, 25]. Although there is more IL-17C and IL-17F in psoriatic lesions, IL-17A is the most biologically active (up to 30-fold more active than IL-17F) [10, 24]. While these three cytokines act on keratinocytes to stimulate production of proinflammatory cytokines and chemokines, the exact role of IL-17C in psoriasis pathogenesis is poorly understood [6, 25]. Despite high levels of IL-17C in psoriatic lesions, IL-17C has less impact on keratinocyte gene expression than IL-17A, IFN-γ, and TNF-α, suggesting that IL-17A is more important than IL-17C in promoting cutaneous inflammation [26].

Therapies targeting IL-17A alone are known to modulate gene expression of various cytokines and chemokines, and effectively clear psoriatic lesions [6, 27–29]. More specifically, 2 weeks of IL-17A inhibition resulted in normalization of 765 genes, whereas TNF-α inhibition resulted in the normalization of far fewer genes (< 200) [30, 31]. In this article, the immunologic role of IL-17 in psoriasis and psoriatic arthritis (PsA) pathogenesis, including its role in innate and adaptive immunity, and the rationale for targeting IL-17A, IL-17F, and IL-17 receptor A in the treatment of psoriasis and PsA, are reviewed.

## Cellular Sources of Interleukin-17 in Innate and Adaptive Immunity

For several years, it was hypothesized that the primary source of IL-17A in psoriasis was T helper 17 (Th17) cells. Specifically, human Th17 cells differentiate from naïve T cells under the influence of TGF-β1 and proinflammatory cytokines (IL-1β, IL-6, and/or IL-21) [32, 33]. Differentiated human Th17 cells are stimulated to produce cytokines by IL-23, which also promotes the survival of Th17 cells [34, 35]. Th17 cells produce a wide variety of cytokines in addition to IL-17A, including IL-17F, IL-21, IL-22, IL-26, and TNF-α [34, 36]. Additionally, increased numbers of Th17 cells are found in the blood and affected skin of patients with psoriasis and in the blood and synovial fluid of patients with PsA [11, 37]. Recently, however, there has been a paradigm shift in the understanding of cellular sources of IL-17A in psoriasis and PsA. Increasingly, data indicate that additional important cellular sources of IL-17A are mast cells, γδ T cells, αβ T cells, and innate lymphoid cells (ILCs; Table 1) [38, 39, 47].

**Table 1:** Cellular sources of IL-17 [38–46]

Cell type	Description
γδ T cells	• Potent source of innate IL-17 produced independently of IL-6 • Properties are similar to Th17 cells (e.g., expression of CCR6, IL-23R, and RORγt); these cells also express TLR1, TLR2, and dectin-1 • Levels of IL-17-producing γδ T cells increase during some types of bacterial infections • Different subsets of γδ T cells in the thymus produce either IL-17 or IFN-γ • Major source of gut-protective IL-17, which acts independently from IL-23
αβ T cells	• Recent data indicate CD4/CD8 double-negative αβ T cells produce IL-17 in psoriatic inflammation • These cells respond to IL-23 to produce IL-17 • These cells likely express RORγt and CCR6
Neutrophils	• Rich source of IL-17 in psoriasis • IL-17 is held and released by neutrophils via extracellular trap formation • Conflicting data have been reported on whether IL-17 mRNA is present in neutrophils
Mast cells	• In response to trauma or infection, preformed inflammatory mediators, including IL-17, are released from mast cells via granulation or mast cell extracellular trap cell death • Mast cells also express IL-17 mRNA and produce IL-17A and IL-17 receptor A
ILC3s	• Subset of ILCs defined by their capacity to produce IL-17A and/or IL-22 • Found in lesional and non-lesional skin, and in peripheral blood in patients with psoriasis, and in synovial fluid in patients with PsA
iNKT cells	• Cells that express a restricted TCR that recognizes glycolipid antigens • May provide an alternative source of IL-17 when IL-6 is not present to stimulate Th17 cells • IL-17^+^ cells express IL-23R and IL-1R1
Adaptive Th17 cells	• A subset of activated CD4^+^ T helper cells that produce high levels of IL-17A, IL-17F, IL-22, and IFN-γ, and express IL-23R • CD4^+^ TCRα/β^+^ Th17 cells are a well-characterized source of IL-17 that play a key role in immune inflammatory responses
Natural Th17 cells	• Subset of thymic Th17 cells that acquire effector function prior to peripheral antigen exposure • These cells have different TCR gene usage and signaling properties compared with conventional Th17 cells
Tc17 cells	• Subset of CD8^+^ cells that produces IL-17 • May play a role in pathogenic skin and joint inflammation in psoriasis and PsA, respectively

*CCR6*, C-C chemokine receptor type 6; *CD*, cluster of cell differentiation; *IFN*, interferon; *IL*, interleukin; *IL-1R1*, interleukin-1 receptor, type 1; *IL-23R*, interleukin-23 receptor; *ILC*, innate lymphoid cell; *iNKT*, invariant natural killer T; *PsA*, psoriatic arthritis; *ROR*, retinoic orphan receptor; *Tc17*, IL-17-expressing CD8+ T cells; *TCR*, T cell receptor; *Th17*, T helper 17; *TLR*, toll-like receptor

It was long thought that neutrophils were an abundant source of IL-17A in psoriasis; however, emerging data indicate that highly purified human neutrophils are not capable of expressing IL-17A or other IL-17 family cytokines in vitro [40, 48–51]. Rather, IL-17A may be released from extracellular neutrophil traps, which are a central function of neutrophil host defense and inflammatory function [40, 41, 52]. Studies on this topic have yielded differing results: (1) retinoic orphan receptor (ROR)γt^+^ neutrophils expressed IL-17 mRNA, and were capable of producing IL-17 [41, 47]; (2) certain populations of bone marrow neutrophils exhibited autocrine IL-17 activity, which was driven by interactions between IL-17A and IL-17RC [53]; and (3) neutrophils in psoriatic lesions produce IL-17 [54]. Regardless of its source, neutrophil-derived IL-17 may be an early target of IL-17A inhibitors as these drugs interrupt neutrophil-keratinocyte crosstalk and disrupt the influx of neutrophils into psoriatic lesions [48].

Similarly, mast cell extracellular trap formation, induced by IL-23 and IL-1β, is associated with the release of IL-17 [40, 47]. When mast cells in healthy skin respond to trauma or microbial infection, preformed inflammatory mediators, including TNF-α, IL-17, and CXCL2, are released via degranulation or mast cell extracellular trap cell death [40]. Mast cells may also express IL-17 mRNA, produce small amounts of IL-17A, and express IL-17 receptor A [42]. Additionally, mast cells can capture, store, and release exogenous IL-17A and trigger the release of IL-17 and IFN-γ from Th1 and Th17 cells by modulating dendritic cell maturation and function [55, 56].

Neutrophils, mast cells, and other innate immune cells are also important in the pathogenesis of PsA [57]. In patients with PsA, high levels of IL-23, IL-17A, and IL-17 receptor A are present in synovial membranes, and resident Th17 cells located in entheses overexpress IL-17 and IL-22, contributing to inflammation and bone remodeling [58, 59]. Additionally, activation of the IL-23/IL-17 axis promotes production of granulocyte-colony stimulating factor, granulocyte-macrophage stimulating factor, and chemokines, including CXCL1, CXCL2, CXCL5, and CXCL8/IL-8, which promote neutrophil recruitment and migration into joint spaces [60]. Mast cell infiltration and IL-17A expression are also observed in spondyloarthritis synovial inflammation, and both mast cells and neutrophils (as opposed to T cells) are major cellular sources of IL-17 in atherosclerosis [40, 61, 62].

Elevated levels of γδ T cells, which can express RORγt, IL-23R, and C-C chemokine receptor type 6 (CCR6), are found in the dermis of psoriatic plaques as well as in the peripheral blood and synovial fluid of patients with PsA; of note, IL-17 signaling was higher in psoriatic lesional skin than in synovial tissue of patients having both skin and joint involvement [12, 63]. Stimulation of these cells with IL-23 results in production of IL-17 and IL-17 expression is observed in synovial tissue of patients with PsA [64–66]. Dermal γδ T cell production of IL-17 is likely independent of αβ T cells; however, a CD4 and CD8 double-negative subset of αβ T cells can produce IL-17 and contribute to psoriatic skin inflammation [39]. In a murine model of psoriasis, a subset of RORγt^+^ γδ T cells form resident-memory cells in skin that rapidly produce large amounts of IL-17A/F [67]. Additionally, CCR6 is a cell surface marker of peripheral IL-17A-expressing γδ T cells [68]. IL-17A in the epidermis can induce keratinocyte expression of chemokine ligand 20 (CCL20), which, in turn, recruits IL-17A-producing CD8+ T cells (Tc17) and CCR6^+^ CD4+ T cells into skin [1, 69–73]. CCR6^+^ cells also migrate to the epidermis or dermal-epidermal junction in response to psoriasis-triggering stimuli in murine models of psoriasis [68, 74]. In human psoriatic lesions, expression of CCR6 and its ligand CCL20 by dendritic cells and T cells has led to a hypothesis that interactions between CCR6 and CCL20 play an important role in crosstalk between dendritic cells and T cells, which ultimately causes T cell activation [75, 76]. Furthermore, synovial fluid of patients with PsA is enriched with CCR6^+^ ILCs [37, 43]. CCR6 is, therefore, being investigated as a possible new target in the treatment of both psoriasis and PsA [68, 75–77].

ILC3s produce IL-17A and are implicated in psoriasis and PsA pathogenesis [43, 78]. Elevated numbers of ILC3s are found in lesional and non-lesional skin of patients with psoriasis, in peripheral blood of patients with psoriasis, and in synovial fluid of patients with PsA [43–45, 78]. ILC3s express high levels of IL-17A, IL-22, CCR6, and natural cytotoxicity receptors, which are all upregulated in psoriatic lesions [43–45, 79]. Expression of IL-17 and IL-22 in ILC3s is specifically dependent on expression and stimulation of RORγt [79, 80]. IL-23 and TNF-α also promote ILC3 differentiation [45, 79]. Interestingly, natural cytotoxicity receptor positive ILC3 levels correlate with psoriasis severity in untreated patients and decrease with anti-TNF-α therapy [45, 79]. Additionally, murine models of psoriasis indicate that ILC3s may be a rich source of non-T cell-derived IL-22 [77, 78].

## Role of Interleukin-17 in the Pathogenesis of Psoriasis and Psoriatic Arthritis

Early studies on the pathogenesis of chronic inflammatory diseases, including rheumatoid arthritis, psoriasis, and inflammatory bowel disease, led to identification of TNF-α as a key trigger of innate inflammatory pathways [31]. Although TNF-α blockers first successfully treated rheumatoid arthritis, they were quickly extended to psoriasis and PsA. Effects of TNF-α inhibition in psoriasis and PsA are complex, because therapeutic benefits likely result from indirect adaptive immune effects on the IL-23/IL-17A axis [31]. Evidence of this indirect effect was observed in clinical trials of etanercept, in which genomic data indicated that etanercept efficacy was dependent on downregulation of IL-17A or IL-17A signaling [31, 81]. The relationship between IL-17 and TNF-α is further complicated as they act synergistically to co-regulate many keratinocyte genes that are highly expressed in psoriatic skin lesions [5]. Together, these findings suggest that IL-17A and TNF-α act through distinct mechanisms to regulate downstream gene expression, with the IL-23/IL-17A axis at the core of psoriasis pathogenesis, and TNF-α playing a more ancillary role in promoting inflammation through synergism with IL-17A and through development and maturation of myeloid dendritic cells [6, 31]. This hypothesis is further supported by evidence that IL-17A inhibition alone is highly effective in psoriasis and PsA in the absence of TNF-α inhibition [7].

**Fig. 1:**
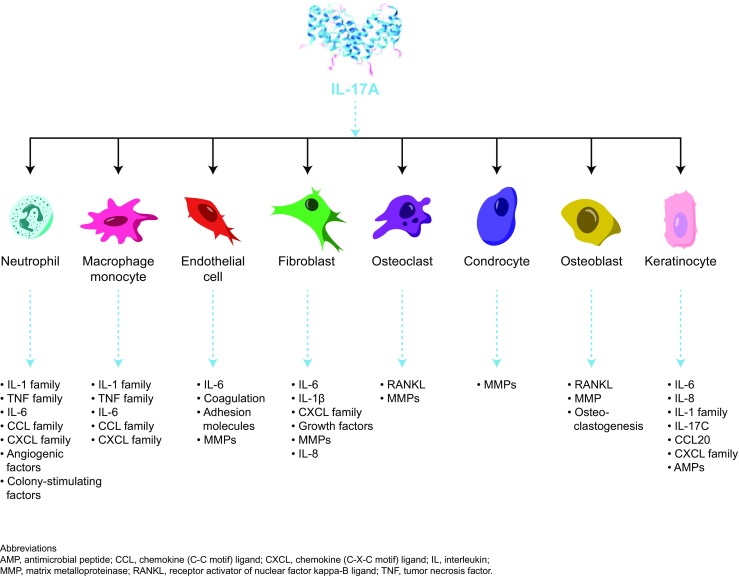
Effects of IL-17 on different cellular targets

IL-23 and IL-17A are key inflammatory cytokines in psoriasis pathogenesis [82, 83]. IL-23 stimulates differentiation, activation, proliferation, and survival of Th17 cells that promote production of effector cytokines such as IL-17A and IL-22, but IL-17 is also produced independently of IL-23 [13, 82, 84–86]. IL-23 injection produces psoriasis-like disease in wild-type mice, but not in *IL17* knockout mice, and IL-23-mediated disease could be blocked in wild-type mice by pretreatment with anti-IL-17A antibodies [82]. This and similar evidence in other IL-23/IL-17-mediated murine disease models indicate that IL-23 is “upstream” of IL-17A, whereas IL-17A, acting “downstream,” directly affects tissue. IL-17A has a range of effects on different cellular targets within the skin and joints by promoting inflammation, coagulation, and bone/joint damage (Fig. 1) [87–89].

Major targets of IL-17 in psoriasis include keratinocytes, endothelial cells, and innate immune cells [89]. In keratinocytes, IL-17 stimulates production of antimicrobial peptides (lipocalin 2, S100A proteins (S100A7, psoriasin), and beta defensins), proinflammatory cytokines and chemokines (IL-1β, TNF-α, IL-6, IL-17C, CXCL1, CXCL3, CXCL5, CXCL8 (IL-8), and CCL20), and proproliferative cytokines (IL-19) [5, 73, 89]. In endothelial cells, IL-17 interacts to promote tissue inflammation and procoagulant activity through upregulation of IL-6, IL-8, and intracellular adhesion molecule-1 [87, 89]. Moreover, IL-17-mediated endothelial dysfunction may contribute to development of cardiovascular comorbidities in psoriasis [89]. Although fibroblasts are not considered disease-relevant/critical target cells, they are capable of sustaining inflammation; an in vitro study showed they produce proinflammatory mediators, including IL-8, IL-1β, and IL-6 and CXCL1, CXCL2, CXCL3, CXCL5, and CXCL6, in response to IL-17 [13]. Lastly, IL-17A has proinflammatory effects on antigen-presenting cells, including macrophages [90].

The centrality of IL-23 and IL-17A to psoriasis and PsA pathogenesis has resulted in many new biologic therapies targeting these cytokines (Table 2) [98, 99]. These drugs, however, can have notable clinical differences related to dosing and safety profiles. Therapies targeting cytokines further upstream in this pathway require less-frequent dosing to maintain efficacy than drugs targeting more downstream cytokines and receptors [100]. IL-23 and IL-12/23 inhibitors (furthest upstream) require maintenance dosing every 8–12 weeks, whereas maintenance dosing with approved IL-17A inhibitors (midstream) is required every 4 weeks, and the IL-17 receptor A antagonist brodalumab (furthest downstream) is administered every 2 weeks [98, 101]. Mechanistic studies indicate that IL-17A has both protective and proinflammatory effects in the gut. There is strong evidence to support the role of γδ T cell-derived IL-17A in the protection of epithelial barriers in the intestinal mucosa; as such, IL-17A blockade may exacerbate inflammatory bowel disease [102–106]. Additionally, IL-17A production has been observed in subpopulations of T regulatory cells, and it is hypothesized that these cells may be protective against inflammatory bowel disease [107, 108]. Pooled safety data from clinical trials of ixekizumab and secukinumab, however, show that exacerbation of inflammatory bowel disease rarely occurs with IL-17A inhibition [109–111]. Nonetheless, IL-17A inhibitors should be used with caution in patients with a history of inflammatory bowel disease [112]. IL-17A blockers are also associated with increased risk for mucocutaneous candidiasis (< 5% of treated patients) because IL-17 is important in the control of *Candida albicans* infections within skin and mucosa [99, 113]. Thus, patients being treated with IL-17A blockers should be screened regularly for signs of mucocutaneous candidiasis; in the small percentage of patients who develop these types of infections, treatment with topical or oral antifungal therapy is generally effective and discontinuation of anti-IL-17A therapy is not necessary [113]. In phase 3 psoriasis trials of brodalumab, psychiatric adverse events, including depression, anxiety, and suicidal ideation and behavior, were observed, suggesting a possible safety concern [114]. However, analysis of data across five clinical trials did not find a causal relationship between treatment with brodalumab and suicidality; rates of adverse events of suicidal ideation and behavior were similar with brodalumab, placebo, and ustekinumab [114]. Patients with psoriasis are known to be at increased risk for psychiatric comorbidities, and all patients with suicidal ideation who received brodalumab had underlying psychiatric disorders or stresses [115]. Of note, similar safety signals have not been observed with either secukinumab or ixekizumab [116].

**Table 2:** Drugs that inhibit IL-23 or IL-17 function

Drug name	Target	FDA approval date and indication
Ustekinumab [91]	p40 subunit (IL-12 and IL-23)	2009 psoriasis, 2013 PsA
Guselkumab [92]	p19 subunit (IL-23)	2017 psoriasis
Tildrakizumab [93]	p19 subunit (IL-23)	2018 psoriasis
Risankizumab [94]	p19 subunit (IL-23)	Not approved
Mirikizumab (NCT03482011)	p19 subunit (IL-23)	Not approved
Secukinumab [27]	IL-17A	2015 psoriasis, 2016 PsA
Ixekizumab [95]	IL-17A	2016 psoriasis, 2017 PsA
Bimekizumab [96]	IL-17A and IL-17F	Not approved
Brodalumab [97]	IL-17RA (IL-17A, IL-17E, IL-17F)	2017 psoriasis

*FDA*, Food and Drug Administration; *IL*, interleukin; *IL-17RA*, IL-17 receptor A; *PsA*, psoriatic arthritis

## Expanding Our Understanding of the Immunologic Role of IL-17

An important issue in managing psoriasis is recurrence of lesions after treatment discontinuation, which is linked with a residual disease genomic profile [117–119]. Relapses may be caused by residual tissue-resident memory T cells not being fully eradicated with anti-TNF therapy. In an etanercept trial, a subset of inflammatory genes that contribute to psoriasis pathogenesis, including IL-12p35, IL-22, IL-17, and IFN-γ, did not return to non-lesional levels [117, 118]. Particularly, clinical recurrences at the same body areas may be determined by the marked presence of IL-17A-producing αβ T cell clones in post-treatment-resolved psoriatic lesional skin, which produce eightfold more IL-17A than αβ T cell clones in healthy skin [120].

Given the critical role of IL-17A in psoriasis pathogenesis, it is not surprising that the IL-17A inhibitors secukinumab and ixekizumab are associated with complete or near complete skin clearance in many patients and have demonstrated efficacy that is superior to many other agents (i.e., TNF-α inhibitors and ustekinumab) [27, 95, 121]. In patients with psoriasis, IL-17A inhibition by secukinumab normalizes levels of dysregulated proteins, including IL-1β, IL-8, IL-1 receptor antagonist, myeloperoxidase, antimicrobial peptides (β-defensin 2 and lipocalin 2), matrix metalloproteinase-1, matrix metalloproteinase-8, matrix metalloproteinase-9, and the chemokines CXCL1, CXCL5, and CCL20 [122, 123]. Secukinumab also decreases mRNA levels of antimicrobial peptides, chemokines, IL-36α, IL-36β, IL-36γ, IL-36RN, IL-17A, and IL-17F [122]. Additionally, ixekizumab normalizes > 3 times more genes than etanercept after 2 weeks [30, 31]. Targeting IL-17 receptor A with brodalumab is also highly effective and inhibits signaling induced by IL-17A, IL-17F, IL-17E (IL-25), and IL-17A/F [97, 124, 125]. Brodalumab also normalizes psoriatic lesional skin transcriptome, the gene expression profile associated with IL-17A, IL-17C, and IL-17F, and reduces IL-23 levels along with keratinocyte-derived mediators of inflammation, including chemokines, IL-36A, and S100s [125]. More recently, bimekizumab, a monoclonal antibody targeting IL-17A and IL-17F, demonstrated high efficacy in psoriasis [96]. Whether this is due to the highly effective blockade of IL-17A or combined effects of blocking 2 IL-17 isoforms is unclear. Interestingly, studies indicate the blockade of both IL-17A and IL-17F decreases inflammation more than the inhibition of IL-17A alone [126–128].

Although the IL-17A gene signature is higher in skin from patients with PsA compared with joints, IL-17A is thought to play a key role in PsA pathogenesis, acting on synovial-like joint fibroblasts, osteoblasts, and osteoclast precursors to promote inflammation and joint damage [12, 129]. Specifically, IL-17A, TNF-α, IL-23, and other inflammatory cytokines activate the innate immune regulators, nuclear factor κB (NFκB), and its receptor activator/ligand (RANK/RANKL). NFκB and RANKL upregulation triggers transcription of genes that promote secretion of bone matrix-degrading enzymes, including matrix metalloproteinase-9, tartrate-resistant acid phosphatase, and cathepsin K [60, 130, 131]. The IL-17A inhibitors secukinumab and ixekizumab are approved for PsA based on phase 3 data (FUTURE 1 and FUTURE 2 for secukinumab, SPIRIT-P1 and SPIRIT-P2 for ixekizumab) [132–135]. These studies demonstrated that treatment with IL-17A blockers improved joint and skin signs and symptoms of PsA, along with physical functioning and quality of life, compared with placebo [132–135]. Finally, a phase 2 study of brodalumab in PsA provided improvements in joint and skin symptoms and physical functioning (with higher doses) compared with placebo [136].

IL-17 also promotes vascular inflammation, endothelial dysfunction, coagulation, thrombosis, and arterial hypertension. Correspondingly, elevated serum IL-17 has been observed in patients with acute myocardial infarction, and monoclonal antibodies that neutralize IL-17 may improve outcomes in patients with psoriasis and/or PsA and comorbid cardiovascular disease [137, 138]. This hypothesis is further supported by a murine model of atherosclerosis; inhibition of IL-17A led to prevention of lesion progression and induction of plaque stabilization in advanced lesions [90]. In a murine model of IL-17A overexpression, neutralization of cytokines downstream of IL-17A improved vascular health [139]. Additionally, anti-IL-17A monoclonal antibodies prevented vascular disease in a murine model of psoriasis [140]. In humans, an acute myocardial infarction registry demonstrated that serum IL-17A below a median of 6.26 pg/mL was associated with higher risk for all-cause mortality and recurrent myocardial infarction, but many patients had IL-17A levels below the assay’s detection limit of 2.5 pg/mL [141]. To more directly study this issue in moderate-to-severe psoriasis, Gelfand and colleagues are assessing whether treatment with secukinumab can lead to improvements in aortic inflammation (VIP-S, NCT02690701), a well-established biomarker of atherosclerotic cardiovascular disease.

## Conclusions

Many cytokines are involved in psoriasis development; however, data identify IL-17A as the major effector cytokine driving pathogenesis. IL-17 is produced by many cell types, acts on a range of cellular targets in tissue and immune cells, and plays important roles in innate and adaptive immunity. Inhibition of IL-17A, IL-17 receptor A, or simultaneous inhibition of IL-17A and IL-17F leads to disruption of signaling pathways critical to the development and maintenance of psoriasis. Accordingly, biologics that target IL-17A function lead to rapid and dramatic improvement of skin and joint symptoms in psoriasis and PsA.

## References

[1] Lowes MA, Suárez-Fariñas M, Krueger JG (2014). Immunology of psoriasis. Annu Rev Immunol.

[2] Nestle FO, Kaplan DH, Barker J (2009). Psoriasis. N Engl J Med.

[3] Boehncke WH, Schön MP (2015). Psoriasis. Lancet.

[4] Chandra A, Ray A, Senapati S, Chatterjee R (2015). Genetic and epigenetic basis of psoriasis pathogenesis. Mol Immunol.

[5] Chiricozzi A, Guttman-Yassky E, Suárez-Fariñas M, Nograles KE, Tian S, Cardinale I, Chimenti S, Krueger JG (2011). Integrative responses to IL-17 and TNF-α in human keratinocytes account for key inflammatory pathogenic circuits in psoriasis. J Invest Dermatol.

[6] Martin DA, Towne JE, Kricorian G, Klekotka P, Gudjonsson JE, Krueger JG, Russell CB (2013). The emerging role of IL-17 in the pathogenesis of psoriasis: preclinical and clinical findings. J Invest Dermatol.

[7] Marinoni B, Ceribelli A, Massarotti MS, Selmi C (2014). The Th17 axis in psoriatic disease: pathogenetic and therapeutic implications. Auto Immun Highlights.

[8] Chiricozzi A, Suárez-Fariñas M, Fuentes-Duculan J, Cueto I, Li K, Tian S, Brodmerkel C, Krueger JG (2016). Increased expression of interleukin-17 pathway genes in nonlesional skin of moderate-to-severe psoriasis vulgaris. Br J Dermatol.

[9] Wang CQ, Suarez-Farinas M, Nograles KE, Mimoso CA, Shrom D, Dow ER, Heffernan MP, Hoffman RW, Krueger JG (2014). IL-17 induces inflammation-associated gene products in blood monocytes, and treatment with ixekizumab reduces their expression in psoriasis patient blood. J Invest Dermatol.

[10] Kirkham BW, Kavanaugh A, Reich K (2014). Interleukin-17A: a unique pathway in immune-mediated diseases: psoriasis, psoriatic arthritis and rheumatoid arthritis. Immunology.

[11] Kagami S, Rizzo HL, Lee JJ, Koguchi Y, Blauvelt A (2010). Circulating Th17, Th22, and Th1 cells are increased in psoriasis. J Invest Dermatol.

[12] Belasco J, Louie JS, Gulati N, Wei N, Nograles K, Fuentes-Duculan J, Mitsui H, Suárez-Fariñas M, Krueger JG (2015). Comparative genomic profiling of synovium versus skin lesions in psoriatic arthritis. Arthritis Rheumatol.

[13] Chiricozzi A, Nograles KE, Johnson-Huang LM, Fuentes-Duculan J, Cardinale I, Bonifacio KM, Gulati N, Mitsui H, Guttman-Yassky E, Suárez-Fariñas M, Krueger JG (2014). IL-17 induces an expanded range of downstream genes in reconstituted human epidermis model. PLoS One.

[14] Lande R, Botti E, Jandus C, Dojcinovic D, Fanelli G, Conrad C, Chamilos G, Feldmeyer L, Marinari B, Chon S, Vence L, Riccieri V, Guillaume P, Navarini AA, Romero P, Costanzo A, Piccolella E, Gilliet M, Frasca L (2014). The antimicrobial peptide LL37 is a T-cell autoantigen in psoriasis. Nat Commun.

[15] Krueger JG (2015). An autoimmune “attack” on melanocytes triggers psoriasis and cellular hyperplasia. J Exp Med.

[16] Arakawa A, Siewert K, Stöhr J, Besgen P, Kim SM, Rühl G, Nickel J, Vollmer S, Thomas P, Krebs S, Pinkert S, Spannagl M, Held K, Kammerbauer C, Besch R, Dornmair K, Prinz JC (2015). Melanocyte antigen triggers autoimmunity in human psoriasis. J Exp Med.

[17] Gaffen SL (2011). Recent advances in the IL-17 cytokine family. Curr Opin Immunol.

[18] Hymowitz SG, Filvaroff EH, Yin JP, Lee J, Cai L, Risser P, Maruoka M, Mao W, Foster J, Kelley RF, Pan G, Gurney AL, de Vos AM, Starovasnik MA (2001). IL-17s adopt a cystine knot fold: structure and activity of a novel cytokine, IL-17F, and implications for receptor binding. EMBO J.

[19] Li H, Chen J, Huang A, Stinson J, Heldens S, Foster J, Dowd P, Gurney AL, Wood WI (2000). Cloning and characterization of IL-17B and IL-17C, two new members of the IL-17 cytokine family. Proc Natl Acad Sci U S A.

[20] Fort MM, Cheung J, Yen D, Li J, Zurawski SM, Lo S, Menon S, Clifford T, Hunte B, Lesley R, Muchamuel T, Hurst SD, Zurawski G, Leach MW, Gorman DM, Rennick DM (2001). IL-25 induces IL-4, IL-5, and IL-13 and Th2-associated pathologies in vivo. Immunity.

[21] Starnes T, Broxmeyer HE, Robertson MJ, Hromas R (2002). Cutting edge: IL-17D, a novel member of the IL-17 family, stimulates cytokine production and inhibits hemopoiesis. J Immunol.

[22] Yang XO, Chang SH, Park H, Nurieva R, Shah B, Acero L, Wang YH, Schluns KS, Broaddus RR, Zhu Z, Dong C (2008). Regulation of inflammatory responses by IL-17F. J Exp Med.

[23] Chang SH, Reynolds JM, Pappu BP, Chen G, Martinez GJ, Dong C (2011). Interleukin-17C promotes Th17 cell responses and autoimmune disease via interleukin-17 receptor E. Immunity.

[24] Johansen C, Usher PA, Kjellerup RB, Lundsgaard D, Iversen L, Kragballe K (2009). Characterization of the interleukin-17 isoforms and receptors in lesional psoriatic skin. Br J Dermatol.

[25] Johnston A, Fritz Y, Dawes SM, Diaconu D, Al-Attar PM, Guzman AM, Chen CS, Fu W, Gudjonsson JE, McCormick TS, Ward NL (2013). Keratinocyte overexpression of IL-17C promotes psoriasiform skin inflammation. J Immunol.

[26] Muromoto R, Hirao T, Tawa K, Hirashima K, Kon S, Kitai Y, Matsuda T (2016). IL-17A plays a central role in the expression of psoriasis signature genes through the induction of IκB-ζ in keratinocytes. Int Immunol.

[27] Langley RG, Elewski BE, Lebwohl M, Reich K, Griffiths CE, Papp K, Puig L, Nakagawa H, Spelman L, Sigurgeirsson B, Rivas E, Tsai TF, Wasel N, Tyring S, Salko T, Hampele I, Notter M, Karpov A, Helou S, Papavassilis C, ERASURE and FIXTURE Study Groups (2014). Secukinumab in plaque psoriasis—results of two phase 3 trials. N Engl J Med.

[28] Gordon KB, Blauvelt A, Papp KA, Langley RG, Luger T, Ohtsuki M, Reich K, Amato D, Ball SG, Braun DK, Cameron GS, Erickson J, Konrad RJ, Muram TM, Nickoloff BJ, Osuntokun OO, Secrest RJ, Zhao F, Mallbris L, Leonardi CL, UNCOVER-1, UNCOVER-2, UNCOVER-3 Study Groups (2016). Phase 3 trials of ixekizumab in moderate-to-severe plaque psoriasis. N Engl J Med.

[29] Hueber W, Patel DD, Dryja T, Wright AM, Koroleva I, Bruin G, Antoni C, Draelos Z, Gold MH, Psoriasis Study G, Durez P, Tak PP, Gomez-Reino JJ, Foster CS, Kim RY, Samson CM, Falk NS, Chu DS, Callanan D, Nguyen QD, Rose K, Haider A, Di Padova F, Rheumatoid Arthritis Study G, Uveitis Study G (2010). Effects of AIN457, a fully human antibody to interleukin-17A, on psoriasis, rheumatoid arthritis, and uveitis. Sci Transl Med.

[30] Krueger JG, Fretzin S, Suárez-Fariñas M, Haslett PA, Phipps KM, Cameron GS, McColm J, Katcherian A, Cueto I, White T, Banerjee S, Hoffman RW (2012). IL-17A is essential for cell activation and inflammatory gene circuits in subjects with psoriasis. J Allergy Clin Immunol.

[31] Zaba LC, Suárez-Fariñas M, Fuentes-Duculan J, Nograles KE, Guttman-Yassky E, Cardinale I, Lowes MA, Krueger JG (2009). Effective treatment of psoriasis with etanercept is linked to suppression of IL-17 signaling, not immediate response TNF genes. J Allergy Clin Immunol.

[32] Volpe E, Servant N, Zollinger R, Bogiatzi SI, Hupe P, Barillot E, Soumelis V (2008). A critical function for transforming growth factor-β, interleukin 23 and proinflammatory cytokines in driving and modulating human T_H_-17 responses. Nat Immunol.

[33] Wei L, Laurence A, Elias KM, O'Shea JJ (2007). IL-21 is produced by Th17 cells and drives IL-17 production in a STAT3-dependent manner. J Biol Chem.

[34] Di Cesare A, Di Meglio P, Nestle FO (2009). The IL-23/Th17 axis in the immunopathogenesis of psoriasis. J Invest Dermatol.

[35] Stritesky GL, Yeh N, Kaplan MH (2008). IL-23 promotes maintenance but not commitment to the Th17 lineage. J Immunol.

[36] Mortezavi M, Ritchlin C (2015). Immunologic advances reveal new targets in psoriasis and psoriatic arthritis. Discov Med.

[37] Benham H, Norris P, Goodall J, Wechalekar MD, FitzGerald O, Szentpetery A, Smith M, Thomas R, Gaston H (2013). Th17 and Th22 cells in psoriatic arthritis and psoriasis. Arthritis Res Ther.

[38] Raychaudhuri SP, Raychaudhuri SK (2017). Mechanistic rationales for targeting interleukin-17A in spondyloarthritis. Arthritis Res Ther.

[39] Ueyama A, Imura C, Fusamae Y, Tsujii K, Furue Y, Aoki M, Suzuki M, Okuda T, Oshima I, Yasui K, Shichijo M, Yamamoto M (2017). Potential role of IL-17-producing CD4/CD8 double negative αβ T cells in psoriatic skin inflammation in a TPA-induced STAT3C transgenic mouse model. J Dermatol Sci.

[40] Lin AM, Rubin CJ, Khandpur R, Wang JY, Riblett M, Yalavarthi S, Villanueva EC, Shah P, Kaplan MJ, Bruce AT (2011). Mast cells and neutrophils release IL-17 through extracellular trap formation in psoriasis. J Immunol.

[41] Keijsers RR, Hendriks AG, van Erp PE, van Cranenbroek B, van de Kerkhof PC, Koenen HJ, Joosten I (2014). In vivo induction of cutaneous inflammation results in the accumulation of extracellular trap-forming neutrophils expressing RORγt and IL-17. J Invest Dermatol.

[42] Mashiko S, Bouguermouh S, Rubio M, Baba N, Bissonnette R, Sarfati M (2015). Human mast cells are major IL-22 producers in patients with psoriasis and atopic dermatitis. J Allergy Clin Immunol.

[43] Leijten EF, van Kempen TS, Boes M, Michels-van Amelsfort JM, Hijnen D, Hartgring SA, van Roon JA, Wenink MH, Radstake TR (2015). Brief report: enrichment of activated group 3 innate lymphoid cells in psoriatic arthritis synovial fluid. Arthritis Rheumatol.

[44] Dyring-Andersen B, Geisler C, Agerbeck C, Lauritsen JP, Gúdjonsdottir SD, Skov L, Bonefeld CM (2014). Increased number and frequency of group 3 innate lymphoid cells in nonlesional psoriatic skin. Br J Dermatol.

[45] Villanova F, Flutter B, Tosi I, Grys K, Sreeneebus H, Perera GK, Chapman A, Smith CH, Di Meglio P, Nestle FO (2014). Characterization of innate lymphoid cells in human skin and blood demonstrates increase of NKp44+ ILC3 in psoriasis. J Invest Dermatol.

[46] Bystrom J, Taher TE, Muhyaddin MS, Clanchy FI, Mangat P, Jawad AS, Williams RO, Mageed RA (2015). Harnessing the therapeutic potential of Th17 cells. Mediat Inflamm.

[47] Keijsers RR, Joosten I, van Erp PE, Koenen HJ, van de Kerkhof PC (2014). Cellular sources of IL-17 in psoriasis: a paradigm shift?. Exp Dermatol.

[48] Reich K, Papp KA, Matheson RT, Tu JH, Bissonnette R, Bourcier M, Gratton D, Kunynetz RA, Poulin Y, Rosoph LA, Stingl G, Bauer WM, Salter JM, Falk TM, Blödorn-Schlicht NA, Hueber W, Sommer U, Schumacher MM, Peters T, Kriehuber E, Lee DM, Wieczorek GA, Kolbinger F, Bleul CC (2015). Evidence that a neutrophil-keratinocyte crosstalk is an early target of IL-17A inhibition in psoriasis. Exp Dermatol.

[49] Tamarozzi F, Wright HL, Thomas HB, Edwards SW, Taylor MJ (2014). A lack of confirmation with alternative assays questions the validity of IL-17A expression in human neutrophils using immunohistochemistry. Immunol Lett.

[50] Tamassia N, Arruda-Silva F, Calzetti F, Lonardi S, Gasperini S, Gardiman E, Bianchetto-Aguilera F, Gatta LB, Girolomoni G, Mantovani A, Vermi W, Cassatella MA (2018). A reappraisal on the potential ability of human neutrophils to express and produce IL-17 family members in vitro: failure to reproducibly detect it. Front Immunol.

[51] Tamassia Nicola, Bianchetto-Aguilera Francisco, Arruda-Silva Fabio, Gardiman Elisa, Gasperini Sara, Calzetti Federica, Cassatella Marco A. (2018). Cytokine production by human neutrophils: Revisiting the “dark side of the moon”. European Journal of Clinical Investigation.

[52] Schön MP, Broekaert SM, Erpenbeck L (2017). Sexy again: the renaissance of neutrophils in psoriasis. Exp Dermatol.

[53] Taylor PR, Roy S, Leal SM, Sun Y, Howell SJ, Cobb BA, Li X, Pearlman E (2014). Activation of neutrophils by autocrine IL-17A-IL-17RC interactions during fungal infection is regulated by IL-6, IL-23, RORγt and dectin-2. Nat Immunol.

[54] Dyring-Andersen B, Honoré TV, Madelung A, Bzorek M, Simonsen S, Clemmensen SN, Clark RA, Borregaard N, Skov L (2017). Interleukin (IL)-17A and IL-22-producing neutrophils in psoriatic skin. Br J Dermatol.

[55] Dudeck A, Suender CA, Kostka SL, von Stebut E, Maurer M (2011). Mast cells promote Th1 and Th17 responses by modulating dendritic cell maturation and function. Eur J Immunol.

[56] Noordenbos T, Blijdorp I, Chen S, Stap J, Mul E, Cañete JD, Lubberts E, Yeremenko N, Baeten D (2016). Human mast cells capture, store, and release bioactive, exogenous IL-17A. J Leukoc Biol.

[57] Lories RJ, de Vlam K (2012). Is psoriatic arthritis a result of abnormalities in acquired or innate immunity?. Curr Rheumatol Rep.

[58] Boutet Marie-Astrid, Nerviani Alessandra, Gallo Afflitto Gabriele, Pitzalis Costantino (2018). Role of the IL-23/IL-17 Axis in Psoriasis and Psoriatic Arthritis: The Clinical Importance of Its Divergence in Skin and Joints. International Journal of Molecular Sciences.

[59] Sakkas LI, Bogdanos DP (2017). Are psoriasis and psoriatic arthritis the same disease? The IL-23/IL-17 axis data. Autoimmun Rev.

[60] Suzuki E, Mellins ED, Gershwin ME, Nestle FO, Adamopoulos IE (2014). The IL-23/IL-17 axis in psoriatic arthritis. Autoimmun Rev.

[61] Noordenbos T, Yeremenko N, Gofita I, van de Sande M, Tak PP, Caňete JD, Baeten D (2012). Interleukin-17-positive mast cells contribute to synovial inflammation in spondylarthritis. Arthritis Rheum.

[62] de Boer OJ, van der Meer JJ, Teeling P, van der Loos CM, Idu MM, van Maldegem F, Aten J, van der Wal AC (2010). Differential expression of interleukin-17 family cytokines in intact and complicated human atherosclerotic plaques. J Pathol.

[63] Guggino G, Ciccia F, Di Liberto D, Lo Pizzo M, Ruscitti P, Cipriani P, Ferrante A, Sireci G, Dieli F, Fournie JJ, Giacomelli R, Triolo G (2016). Interleukin (IL)-9/IL-9R axis drives γδ T cells activation in psoriatic arthritis patients. Clin Exp Immunol.

[64] Cai Y, Shen X, Ding C, Qi C, Li K, Li X, Jala VR, Zhang HG, Wang T, Zheng J, Yan J (2011). Pivotal role of dermal IL-17-producing γδ T cells in skin inflammation. Immunity.

[65] O'Brien RL, Born WK (2015). Dermal γδ T cells—what have we learned?. Cell Immunol.

[66] van Baarsen LG, Lebre MC, van der Coelen D, Aarrass S, Tang MW, Ramwadhdoebe TH, Gerlag DM, Tak PP (2014). Heterogeneous expression pattern of interleukin 17A (IL-17A), IL-17F and their receptors in synovium of rheumatoid arthritis, psoriatic arthritis and osteoarthritis: possible explanation for nonresponse to anti-IL-17 therapy?. Arthritis Res Ther.

[67] Hartwig T, Pantelyushin S, Croxford AL, Kulig P, Becher B (2015). Dermal IL-17-producing γδ T cells establish long-lived memory in the skin. Eur J Immunol.

[68] Mabuchi T, Takekoshi T, Hwang ST (2011). Epidermal CCR6^+^ γδ T cells are major producers of IL-22 and IL-17 in a murine model of psoriasiform dermatitis. J Immunol.

[69] Res PC, Piskin G, de Boer OJ, van der Loos CM, Teeling P, Bos JD, Teunissen MB (2010). Overrepresentation of IL-17A and IL-22 producing CD8 T cells in lesional skin suggests their involvement in the pathogenesis of psoriasis. PLoS One.

[70] Ortega C, Fernández-A S, Carrillo JM, Romero P, Molina IJ, Moreno JC, Santamaria M (2009). IL-17-producing CD8^+^ T lymphocytes from psoriasis skin plaques are cytotoxic effector cells that secrete Th17-related cytokines. J Leukoc Biol.

[71] Hijnen D, Knol EF, Gent YY, Giovannone B, Beijn SJ, Kupper TS, Bruijnzeel-Koomen CA, Clark RA (2013). CD8^+^ T cells in the lesional skin of atopic dermatitis and psoriasis patients are an important source of IFN-ɣ, IL-13, IL-17, and IL-22. J Invest Dermatol.

[72] Lowes MA, Kikuchi T, Fuentes-Duculan J, Cardinale I, Zaba LC, Haider AS, Bowman EP, Krueger JG (2008). Psoriasis vulgaris lesions contain discrete populations of Th1 and Th17 T cells. J Invest Dermatol.

[73] Harper EG, Guo C, Rizzo H, Lillis JV, Kurtz SE, Skorcheva I, Purdy D, Fitch E, Iordanov M, Blauvelt A (2009). Th17 cytokines stimulate CCL20 expression in keratinocytes in vitro and in vivo: implications for psoriasis pathogenesis. J Invest Dermatol.

[74] Mabuchi T, Singh TP, Takekoshi T, Jia GF, Wu X, Kao MC, Weiss I, Farber JM, Hwang ST (2013). CCR6 is required for epidermal trafficking of γδ-T cells in an IL-23-induced model of psoriasiform dermatitis. J Invest Dermatol.

[75] Kim TG, Jee H, Fuentes-Duculan J, Wu WH, Byamba D, Kim DS, Kim DY, Lew DH, Yang WI, Krueger JG, Lee MG (2014). Dermal clusters of mature dendritic cells and T cells are associated with the CCL20/CCR6 chemokine system in chronic psoriasis. J Invest Dermatol.

[76] Hedrick MN, Lonsdorf AS, Hwang ST, Farber JM (2010). CCR6 as a possible therapeutic target in psoriasis. Expert Opin Ther Targets.

[77] Hedrick MN, Lonsdorf AS, Shirakawa AK, Richard Lee CC, Liao F, Singh SP, Zhang HH, Grinberg A, Love PE, Hwang ST, Farber JM (2009). CCR6 is required for IL-23-induced psoriasis-like inflammation in mice. J Clin Invest.

[78] Ward NL, Umetsu DT (2014). A new player on the psoriasis block: IL-17A- and IL-22-producing innate lymphoid cells. J Invest Dermatol.

[79] Teunissen MBM, Munneke JM, Bernink JH, Spuls PI, Res PCM, Te Velde A, Cheuk S, Brouwer MWD, Menting SP, Eidsmo L, Spits H, Hazenberg MD, Mjösberg J (2014). Composition of innate lymphoid cell subsets in the human skin: enrichment of NCR^+^ ILC3 in lesional skin and blood of psoriasis patients. J Invest Dermatol.

[80] Tait Wojno ED, Artis D (2012). Innate lymphoid cells: balancing immunity, inflammation, and tissue repair in the intestine. Cell Host Microbe.

[81] Johnston A, Guzman AM, Swindell WR, Wang F, Kang S, Gudjonsson JE (2014). Early tissue responses in psoriasis to the antitumour necrosis factor-α biologic etanercept suggest reduced interleukin-17 receptor expression and signalling. Br J Dermatol.

[82] Rizzo HL, Kagami S, Phillips KG, Kurtz SE, Jacques SL, Blauvelt A (2011). IL-23-mediated psoriasis-like epidermal hyperplasia is dependent on IL-17A. J Immunol.

[83] Puig L (2017). The role of IL 23 in the treatment of psoriasis. Expert Rev Clin Immunol.

[84] Fitch E, Harper E, Skorcheva I, Kurtz SE, Blauvelt A (2007). Pathophysiology of psoriasis: recent advances on IL-23 and Th17 cytokines. Curr Rheumatol Rep.

[85] St Leger AJ, Hansen AM, Karauzum H, Horai R, Yu CR, Laurence A, Mayer-Barber KD, Silver P, Villasmil R, Egwuagu C, Datta SK, Caspi RR (2018). STAT-3-independent production of IL-17 by mouse innate-like alphaβ T cells controls ocular infection. J Exp Med.

[86] Yoshiga Y, Goto D, Segawa S, Ohnishi Y, Matsumoto I, Ito S, Tsutsumi A, Taniguchi M, Sumida T (2008). Invariant NKT cells produce IL-17 through IL-23-dependent and -independent pathways with potential modulation of Th17 response in collagen-induced arthritis. Int J Mol Med.

[87] Miossec P, Kolls JK (2012). Targeting IL-17 and T_H_17 cells in chronic inflammation. Nat Rev Drug Discov.

[88] Zenobia C, Hajishengallis G (2015). Basic biology and role of interleukin-17 in immunity and inflammation. Periodontol.

[89] Chiricozzi A, Krueger JG (2013). IL-17 targeted therapies for psoriasis. Expert Opin Investig Drugs.

[90] Erbel C, Akhavanpoor M, Okuyucu D, Wangler S, Dietz A, Zhao L, Stellos K, Little KM, Lasitschka F, Doesch A, Hakimi M, Dengler TJ, Giese T, Blessing E, Katus HA, Gleissner CA (2014). IL-17A influences essential functions of the monocyte/macrophage lineage and is involved in advanced murine and human atherosclerosis. J Immunol.

[91] Leonardi CL, Kimball AB, Papp KA, Yeilding N, Guzzo C, Wang Y, Li S, Dooley LT, Gordon KB, Phoenix Study Investigators (2008). Efficacy and safety of ustekinumab, a human interleukin-12/23 monoclonal antibody, in patients with psoriasis: 76-week results from a randomised, double-blind, placebo-controlled trial (PHOENIX 1). Lancet.

[92] Blauvelt A, Papp KA, Griffiths CE, Randazzo B, Wasfi Y, Shen YK, Li S, Kimball AB (2017). Efficacy and safety of guselkumab, an anti-interleukin-23 monoclonal antibody, compared with adalimumab for the continuous treatment of patients with moderate to severe psoriasis: results from the phase III, double-blinded, placebo- and active comparator-controlled VOYAGE 1 trial. J Am Acad Dermatol.

[93] Reich K, Papp KA, Blauvelt A, Tyring SK, Sinclair R, Thaçi D, Nograles K, Mehta A, Cichanowitz N, Li Q, Liu K, La Rosa C, Green S, Kimball AB (2017). Tildrakizumab versus placebo or etanercept for chronic plaque psoriasis (reSURFACE 1 and reSURFACE 2): results from two randomised controlled, phase 3 trials. Lancet.

[94] Papp KA, Blauvelt A, Bukhalo M, Gooderham M, Krueger JG, Lacour JP, Menter A, Philipp S, Sofen H, Tyring S, Berner BR, Visvanathan S, Pamulapati C, Bennett N, Flack M, Scholl P, Padula SJ (2017). Risankizumab versus ustekinumab for moderate-to-severe plaque psoriasis. N Engl J Med.

[95] Griffiths CE, Reich K, Lebwohl M, van de Kerkhof P, Paul C, Menter A, Cameron GS, Erickson J, Zhang L, Secrest RJ, Ball S, Braun DK, Osuntokun OO, Heffernan MP, Nickoloff BJ, Papp K, UNCOVER-2 and UNCOVER-3 Investigators (2015). Comparison of ixekizumab with etanercept or placebo in moderate-to-severe psoriasis (UNCOVER-2 and UNCOVER-3): results from two phase 3 randomised trials. Lancet.

[96] Papp KA, Merola JF, Gottlieb AB, Griffiths CEM, Cross N, Peterson L, Cioffi C, Blauvelt A (2018). Dual neutralization of both interleukin 17A and interleukin 17F with bimekizumab in patients with psoriasis: results from BE ABLE 1, a 12-week randomized, double-blinded, placebo-controlled phase 2b trial. J Am Acad Dermatol.

[97] Lebwohl M, Strober B, Menter A, Gordon K, Weglowska J, Puig L, Papp K, Spelman L, Toth D, Kerdel F, Armstrong AW, Stingl G, Kimball AB, Bachelez H, Wu JJ, Crowley J, Langley RG, Blicharski T, Paul C, Lacour JP, Tyring S, Kircik L, Chimenti S, Callis Duffin K, Bagel J, Koo J, Aras G, Li J, Song W, Milmont CE, Shi Y, Erondu N, Klekotka P, Kotzin B, Nirula A (2015). Phase 3 studies comparing brodalumab with ustekinumab in psoriasis. N Engl J Med.

[98] Gaspari AA, Tyring S (2015). New and emerging biologic therapies for moderate-to-severe plaque psoriasis: mechanistic rationales and recent clinical data for IL-17 and IL-23 inhibitors. Dermatol Ther.

[99] Blauvelt A, Lebwohl MG, Bissonnette R (2015). IL-23/IL-17A dysfunction phenotypes inform possible clinical effects from anti-IL-17A therapies. J Invest Dermatol.

[100] Girolomoni G, Strohal R, Puig L, Bachelez H, Barker J, Boehncke WH, Prinz JC (2017). The role of IL-23 and the IL-23/TH 17 immune axis in the pathogenesis and treatment of psoriasis. J Eur Acad Dermatol Venereol.

[101] Nakamura M, Lee K, Jeon C, Sekhon S, Afifi L, Yan D, Lee K, Bhutani T (2017). Guselkumab for the treatment of psoriasis: a review of phase III trials. Dermatol Ther (Heidelb).

[102] O'Connor W, Kamanaka M, Booth CJ, Town T, Nakae S, Iwakura Y, Kolls JK, Flavell RA (2009). A protective function for interleukin 17A in T cell-mediated intestinal inflammation. Nat Immunol.

[103] Hueber W, Sands BE, Lewitzky S, Vandemeulebroecke M, Reinisch W, Higgins PD, Wehkamp J, Feagan BG, Yao MD, Karczewski M, Karczewski J, Pezous N, Bek S, Bruin G, Mellgard B, Berger C, Londei M, Bertolino AP, Tougas G, Travis SP, Secukinumab in Crohn’s Disease Study Group (2012). Secukinumab, a human anti-IL-17A monoclonal antibody, for moderate to severe Crohn's disease: unexpected results of a randomised, double-blind placebo-controlled trial. Gut.

[104] Xu XR, Liu CQ, Feng BS, Liu ZJ (2014). Dysregulation of mucosal immune response in pathogenesis of inflammatory bowel disease. World J Gastroenterol.

[105] Targan SR, Feagan B, Vermeire S, Panaccione R, Melmed GY, Landers C, Li D, Russell C, Newmark R, Zhang N, Chon Y, Hsu YH, Lin SL, Klekotka P (2016). A randomized, double-blind, placebo-controlled phase 2 study of brodalumab in patients with moderate-to-severe Crohn's disease. Am J Gastroenterol.

[106] Lee JS, Tato CM, Joyce-Shaikh B, Gulen MF, Cayatte C, Chen Y, Blumenschein WM, Judo M, Ayanoglu G, McClanahan TK, Li X, Cua DJ (2015). Interleukin-23-independent IL-17 production regulates intestinal epithelial permeability. Immunity.

[107] Marwaha AK, Leung NJ, McMurchy AN, Levings MK (2012). TH17 cells in autoimmunity and immunodeficiency: protective or pathogenic?. Front Immunol.

[108] Li L, Boussiotis VA (2013). The role of IL-17-producing Foxp3+ CD4+ T cells in inflammatory bowel disease and colon cancer. Clin Immunol.

[109] Blauvelt A (2016). Safety of secukinumab in the treatment of psoriasis. Expert Opin Drug Saf.

[110] van de Kerkhof PC, Griffiths CE, Reich K, Leonardi CL, Blauvelt A, Tsai TF, Gong Y, Huang J, Papavassilis C, Fox T (2016). Secukinumab long-term safety experience: a pooled analysis of 10 phase II and III clinical studies in patients with moderate to severe plaque psoriasis. J Am Acad Dermatol.

[111] Reich K, Leonardi C, Langley RG, Warren RB, Bachelez H, Romiti R, Ohtsuki M, Xu W, Acharya N, Solotkin K, Colombel JF, Hardin DS (2017). Inflammatory bowel disease among patients with psoriasis treated with ixekizumab: a presentation of adjudicated data from an integrated database of 7 randomized controlled and uncontrolled trials. J Am Acad Dermatol.

[112] Hohenberger M, Cardwell LA, Oussedik E, Feldman SR (2018). Interleukin-17 inhibition: role in psoriasis and inflammatory bowel disease. J Dermatolog Treat.

[113] Armstrong AW, Bukhalo M, Blauvelt A (2016). A clinician's guide to the diagnosis and treatment of candidiasis in patients with psoriasis. Am J Clin Dermatol.

[114] Lebwohl MG, Papp KA, Marangell LB, Koo J, Blauvelt A, Gooderham M, Wu JJ, Rastogi S, Harris S, Pillai R, Israel RJ (2018). Psychiatric adverse events during treatment with brodalumab: analysis of psoriasis clinical trials. J Am Acad Dermatol.

[115] Rieder EA (2018). In response to Lebwohl et al, “psychiatric adverse events during treatment with brodalumab: analysis of psoriasis clinical trials”. J Am Acad Dermatol.

[116] Chiricozzi A, Romanelli M, Saraceno R, Torres T (2016). No meaningful association between suicidal behavior and the use of IL-17A-neutralizing or IL-17RA-blocking agents. Expert Opin Drug Saf.

[117] Clark RA (2011). Gone but not forgotten: lesional memory in psoriatic skin. J Invest Dermatol.

[118] Suárez-Fariñas M, Fuentes-Duculan J, Lowes MA, Krueger JG (2011). Resolved psoriasis lesions retain expression of a subset of disease-related genes. J Invest Dermatol.

[119] Johnson-Huang LM, Pensabene CA, Shah KR, Pierson KC, Kikuchi T, Lentini T, Gilleaudeau P, Sullivan-Whalen M, Cueto I, Khatcherian A, Hyder LA, Suárez-Fariñas M, Krueger JG, Lowes MA (2012). Post-therapeutic relapse of psoriasis after CD11a blockade is associated with T cells and inflammatory myeloid DCs. PLoS One.

[120] Matos TR, O'Malley JT, Lowry EL, Hamm D, Kirsch IR, Robins HS, Kupper TS, Krueger JG, Clark RA (2017). Clinically resolved psoriatic lesions contain psoriasis-specific IL-17-producing αβ T cell clones. J Clin Invest.

[121] Blauvelt A, Reich K, Tsai TF, Tyring S, Vanaclocha F, Kingo K, Ziv M, Pinter A, Vender R, Hugot S, You R, Milutinovic M, Thaçi D (2017). Secukinumab is superior to ustekinumab in clearing skin of subjects with moderate-to-severe plaque psoriasis up to 1 year: results from the CLEAR study. J Am Acad Dermatol.

[122] Kolbinger F, Loesche C, Valentin MA, Jiang X, Cheng Y, Jarvis P, Peters T, Calonder C, Bruin G, Polus F, Aigner B, Lee DM, Bodenlenz M, Sinner F, Pieber TR, Patel DD (2017). β-Defensin 2 is a responsive biomarker of IL-17A-driven skin pathology in patients with psoriasis. J Allergy Clin Immunol.

[123] Loesche C, Kolbinger F, Valentin MA, Jarvis P, Ceci M, Wieczorek G, Khokhlovich E, Koroleva I, Bruin G, Sinner F, Aigner B, Patel DD (2016). Interleukin-17A blockade with secukinumab results in decreased neutrophil infiltration in psoriasis: minimally-invasive measurement by tape stripping. Adv Precision Med.

[124] Nirula A, Nilsen J, Klekotka P, Kricorian G, Erondu N, Towne JE, Russell CB, Martin DA, Budelsky AL (2016). Effect of IL-17 receptor a blockade with brodalumab in inflammatory diseases. Rheumatology (Oxford).

[125] Russell CB, Rand H, Bigler J, Kerkof K, Timour M, Bautista E, Krueger JG, Salinger DH, Welcher AA, Martin DA (2014). Gene expression profiles normalized in psoriatic skin by treatment with brodalumab, a human anti-IL-17 receptor monoclonal antibody. J Immunol.

[126] Maroof A, Okoye R, Smallie T, Baeten D, Archer S, Simpson C, Griffiths M, Shaw S (2017) Bimekizumab dual inhibition of IL-17A and IL-17F provides evidence of IL-17F contribution to chronic inflammation in disease-relevant cells [abstract]. Arthritis Rheumatol 69(Suppl 10):Abstract 1571

[127] Maroof A, Baeten D, Archer S, Griffiths M, Shaw S (2017). IL-17F contributes to human chronic inflammation in synovial tissue: preclinical evidence with dual IL-17a and IL-17F inhibition with bimekizumab in psoriatic arthritis. Ann Rheum Dis.

[128] Maroof A, Smallie T, Archer S, Simpson C, Griffiths M, Baeten D, Shaw S (2017). Dual IL-17A and IL-17F inhibition with bimekizumab provides evidence for IL-17F contribution in immune-mediated inflammatory skin response [abstract]. J Invest Dermatol.

[129] de Vlam K, Gottlieb AB, Mease PJ (2014). Current concepts in psoriatic arthritis: pathogenesis and management. Acta Derm Venereol.

[130] O'Rielly DD, Rahman P (2015). Genetic, epigenetic and pharmacogenetic aspects of psoriasis and psoriatic arthritis. Rheum Dis Clin N Am.

[131] Wang EA, Suzuki E, Maverakis E, Adamopoulos IE (2017). Targeting IL-17 in psoriatic arthritis. Eur J Rheumatol.

[132] Mease PJ, McInnes IB, Kirkham B, Kavanaugh A, Rahman P, van der Heijde D, Landewé R, Nash P, Pricop L, Yuan J, Richards HB, Mpofu S, FUTURE 1 Study Group (2015). Secukinumab inhibition of interleukin-17A in patients with psoriatic arthritis. N Engl J Med.

[133] McInnes IB, Mease PJ, Kirkham B, Kavanaugh A, Ritchlin CT, Rahman P, van der Heijde D, Landewé R, Conaghan PG, Gottlieb AB, Richards H, Pricop L, Ligozio G, Patekar M, Mpofu S, FUTURE 2 Study Group (2015). Secukinumab, a human anti-interleukin-17A monoclonal antibody, in patients with psoriatic arthritis (FUTURE 2): a randomised, double-blind, placebo-controlled, phase 3 trial. Lancet.

[134] Mease PJ, van der Heijde D, Ritchlin CT, Okada M, Cuchacovich RS, Shuler CL, Lin CY, Braun DK, Lee CH, Gladman DD, SPIRIT-P1 Study Group (2017). Ixekizumab, an interleukin-17A specific monoclonal antibody, for the treatment of biologic-naive patients with active psoriatic arthritis: results from the 24-week randomised, double-blind, placebo-controlled and active (adalimumab)-controlled period of the phase III trial SPIRIT-P1. Ann Rheum Dis.

[135] Nash P, Kirkham B, Okada M, Rahman P, Combe B, Burmester GR, Adams DH, Kerr L, Lee C, Shuler CL, Genovese M, SPIRIT-P2 Study Group (2017). Ixekizumab for the treatment of patients with active psoriatic arthritis and an inadequate response to tumour necrosis factor inhibitors: results from the 24-week randomised, double-blind, placebo-controlled period of the SPIRIT-P2 phase 3 trial. Lancet.

[136] Mease PJ, Genovese MC, Greenwald MW, Ritchlin CT, Beaulieu AD, Deodhar A, Newmark R, Feng J, Erondu N, Nirula A (2014). Brodalumab, an anti-IL17RA monoclonal antibody, in psoriatic arthritis. N Engl J Med.

[137] Mitra A, Raychaudhuri SK, Raychaudhuri SP (2014). IL-17 and IL-17R: an auspicious therapeutic target for psoriatic disease. Actas Dermosifiliogr.

[138] Hashmi S, Zeng QT (2006). Role of interleukin-17 and interleukin-17-induced cytokines interleukin-6 and interleukin-8 in unstable coronary artery disease. Coron Artery Dis.

[139] Karbach S, Croxford AL, Oelze M, Schuler R, Minwegen D, Wegner J, Koukes L, Yogev N, Nikolaev A, Reißig S, Ullmann A, Knorr M, Waldner M, Neurath MF, Li H, Wu Z, Brochhausen C, Scheller J, Rose-John S, Piotrowski C, Bechmann I, Radsak M, Wild P, Daiber A, von Stebut E, Wenzel P, Waisman A, Münzel T (2014). Interleukin 17 drives vascular inflammation, endothelial dysfunction, and arterial hypertension in psoriasis-like skin disease. Arterioscler Thromb Vasc Biol.

[140] Li Y, Golden JB, Camhi MI, Zhang X, Fritz Y, Diaconu D, Ivanco TL, Simon DI, Kikly K, McCormick TS, Wang Y, Ward NL (2018). Protection from psoriasis-related thrombosis after inhibition of IL-23 or IL-17A. J Invest Dermatol.

[141] Simon T, Taleb S, Danchin N, Laurans L, Rousseau B, Cattan S, Montely JM, Dubourg O, Tedgui A, Kotti S, Mallat Z (2013). Circulating levels of interleukin-17 and cardiovascular outcomes in patients with acute myocardial infarction. Eur Heart J.

